# Multidimensional Evaluation of Continuous Positive Airway Pressure (CPAP) Treatment for Sleep Apnea in Different Clusters of Couples

**DOI:** 10.3390/jcm9061658

**Published:** 2020-06-01

**Authors:** Monique Mendelson, Thibaut Gentina, Elodie Gentina, Renaud Tamisier, Jean-Louis Pépin, Sébastien Bailly

**Affiliations:** 1 HP2 laboratory, INSERM U1042, University Grenoble Alpes, 38000 Grenoble, France; rtamisier@chu-grenoble.fr (R.T.); jpepin@chu-grenoble.fr (J.-L.P.); sbailly@chu-grenoble.fr (S.B.); 2 Ramsay General Healthcare, 59800 Lille, France; docteur.gentina@gmail.com; 3 IESEG School of Management, 3 rue de la Digue, 59000 Lille, France; elogentina@hotmail.com

**Keywords:** obstructive sleep apnea, continuous positive airway pressure, adherence, marital quality, latent class analysis

## Abstract

Continuous positive airway pressure (CPAP) is the most efficient treatment of obstructive sleep apnea (OSA). Little is known about the impact of spousal relationship profiles on CPAP adherence. We aimed to identify clusters of couples of OSA patients, and their association with CPAP adherence 120 days after CPAP initiation. In a multicenter prospective study, OSA patients recently prescribed CPAP were enrolled with their spouses. Data about spousal relationships were collected at inclusion and at day 120. Latent class analysis was performed to determine homogeneous groups of spousal relationships. The 290 participants were predominantly males (77%), median age was 53 years and interquartile range (IQR) 46–62, median body mass index (BMI) was 32 kg/m^2^ (IQR: 28.6–35.9) and median apnea–hypopnea index: 43 events per hour (IQR: 33–58). Three couple clusters were identified: (1) older retired couples, (2) young working couples, and (3) mature active couples. Patients in the older retired couples cluster presented the highest CPAP adherence (*p* < 0.01) independently of initial complaints, OSA severity, and degree of improvement under CPAP. In a large cohort of OSA patients in whom clusters of couples were determined, there was a significant difference in CPAP adherence at day-120 after CPAP initiation.

## 1. Introduction

Obstructive sleep apnea (OSA) is a major health concern, affecting nearly 1 billion individuals worldwide with multi-organ consequences that result in considerable economic and social burdens [1,2]. Continuous positive airway pressure (CPAP) is the first line treatment of OSA and is effective for alleviating symptoms, restoring neurocognitive function, and improving quality of life [3]. Although short-term CPAP adherence is good [4], suboptimal adherence to CPAP develops on the long term with only ∼50% using CPAP >4 h per night at 1-year in most trials [5,6,7]. Improvements in subjective and objective outcomes are greater with higher use (i.e., goal of >4 h use per night) [8,9,10].

For patients with chronic disease, “spouses” (refers to all domestic partners in the present manuscript) are frequently described as the greatest source of social support for both the physical and emotional aspects of illness [11]. Spouses can also influence patients’ health behaviors and be an integral component to successful adherence to interventions.

Due to the dyadic (i.e., pairing of two individuals) nature of sleep for adults living with spouses, the effects of OSA and its treatment expand beyond the individual patient. Furthermore, results from studies examining facilitators and barriers to CPAP use perceived by patients have suggested that spouses play an important role [12].

Little is known about the impact of spousal involvement on CPAP adherence. We recently published a study in OSA patients examining marital quality, partner’s engagement, and the characteristics of couples. This study showed that the spouse’s engagement mediates CPAP adherence [13].

The aim of the present study was to expand data from this previous study [13] by identifying specific clusters of couples and their association with CPAP adherence 120 days after CPAP initiation.

## 2. Methods

### 2.1. Study Participants

Participants were recruited consecutively from May 2015 to December 2016, from eight private sleep centers in France. We used a definition of a couple that relies on the following three criteria [14]: (1) being over 18 years of age; (2) sharing the same main residence for at least one year; and (3) declaring to be living as a couple or being married, or living under a civil or common-law union. Included participants were patients newly diagnosed with OSA without any previous experience of CPAP usage. Standardized procedures for CPAP initiation and follow-up were established by the same homecare provider (Elia Medical, Mons En Baroeul, France), thus guaranteeing homogeneous practices for follow-up. We excluded patients with neurocognitive disorders or language fluency problems making them unable to complete study questionnaires. All patients provided written informed consent before participation, and the protocol was approved by the regional ethics Committee (Nord Ouest IV 013 A01842 43).

### 2.2. Assessments

OSA diagnosis was based on a full in-laboratory polysomnography (PSG) or a home sleep test (HST). Apneas, hypopneas, apnea–hypopnea index (AHI), and micro-arousals were scored according to international recommendations [15]. Relevant socio-demographic variables, Epworth Sleepiness Score (ESS), and the Quebec Sleep Questionnaire (QSQ) [16] measuring patient reported disease-specific health-related quality of life, were collected.

At CPAP initiation, without the presence of their spouse, patients evaluated their subjective view of their marital relationship using the Quality of Marriage Index (QMI) [17]. Additional items about the relationship including duration, family context, and sleeping arrangements completed this questionnaire.

Questionnaires were repeated 120 days after CPAP initiation.

The dyadic adjustment scale (DAS) [18] and a quality of marriage index was used to assess the spousal relationship. Spousal involvement in CPAP use was measured by using 23 items from the coping dyadic scale based on the frequency with which the spouse/partner is involved in dealing problems that he/she experiences as part of OSA management [19]. Consistent with Baron et al.’s study [20], we slightly modified the items from the original scale [19] in order to focus on CPAP use. Participants evaluated the extent to which their spouse is involved in the behaviors to encourage patients to use their CPAP machine during the last month on a scale from 1 (not at all) to 5 (extreme). The questionnaire included the three dimensions of the dyadic coping scale: pressure, collaboration, and support.

### 2.3. CPAP Prescription, Initiation and Follow-Up

CPAP was prescribed to OSA patients with AHI > 30 events per hour, or an AHI between 15 and 30 events per hour with symptoms and/or co-morbidities following the rules of the French national consensus statement. Before starting CPAP, patients benefited from a standardized one-hour educational program including a 10-min video about OSA definitions, symptoms, and the benefits of CPAP usage [21]. Objective CPAP adherence data were downloaded at day 120 from the CPAP device’s software (ResScan ResMed, San Diego, CA, USA).

### 2.4. Statistical Analysis

Data were described by using median and interquartile range (Q1, Q3) for quantitative variables and frequency and percentage for qualitative variables. A latent class analysis (LCA) [22] was used to identify homogenous clusters of spousal relationships in 290 patients. LCA is a probabilistic clustering method which allows homogeneous sub-groups of phenotypes, called latent classes, to be identified from a larger heterogeneous population. Patients are classified into clusters based on their higher probability of belonging to one cluster than to another, which is directly estimated by the model. The VarSelLCM R package was used for LCA [23]. This R package considers missing data in the clustering process. The following variables corresponding to the spousal relationship were considered for the clustering: patient gender, age (defined as the patient’s age), marital situation (married, common-law or civil union), presence of children at home, same bed time, shared bedroom, number of years together, and professional activity (active, retired, unemployed). The optimal number of clusters was defined by the integrated completed likelihood criterion. Clusters were compared using the non-parametric Kruskall–Wallis test for quantitative variables and the Chi-square test for qualitative variables. Multivariable mixed linear regressions with a random effect on patient were performed to investigate the association between spousal clusters and quantitative outcomes at day 120 (CPAP adherence and ESS score) by adjusting on patient baseline variables including age, body mass index (BMI), gender, AHI, Epworth sleepiness score, type of CPAP mask, and type of CPAP. Statistical analyses were performed with both SAS v9.4 (SAS Institute Inc., Cary, NC, USA) and R v3.6.1 software (R Foundation for Statistical Computing, Vienna, Austria).

## 3. Results

### 3.1. Patient and Couple Cluster Characteristics

The 290 OSA patients were predominantly males (77%), the median age was 53 years (IQR: 46–62), median BMI: 32 kg/m^2^ (IQR: 28.6–35.9) and median apnea–hypopnea index was 43 events per hour (IQR: 33–58; Table 1).

The majority of couples were married (246, 85%) and had been living together for a median of 25 years (IQR: 13–35.5). The spouse participated in the CPAP initiation session in 63% of cases. A minority of patients had night-time jobs (*n* = 20, 6.9%). The majority of couples had at least one child (241, 83%), and 188 couples had two or more children (65.3%). Most couples shared a bedroom (*n* = 210, 72.4%) and the mattress width was 140 cm for 112 couples (42%).

### 3.2. Cluster Analysis

Three distinct couple clusters were identified using latent class analysis. Baseline patient characteristics based on the different clusters of couples are presented in Table 1.

**Figure 1:**
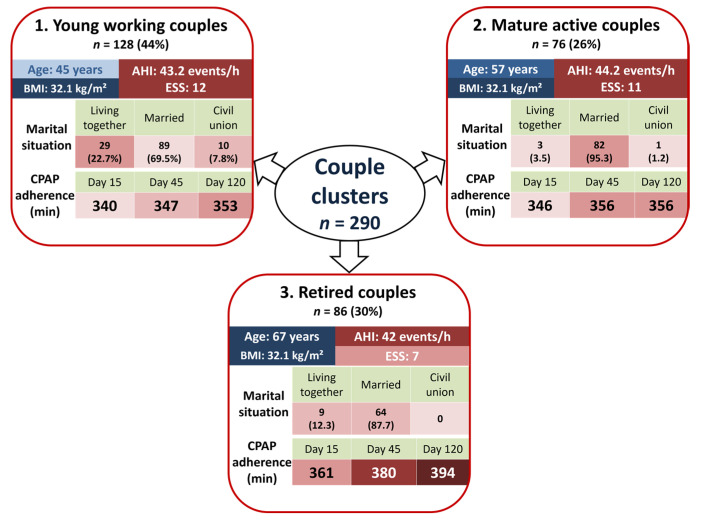
Representation of the three couple clusters. Abbreviations: BMI: body mass index, CPAP: continuous positive airway pressure; AHI: apnea-hypopnea index; ESS: Epworth sleepiness score.

Each couple cluster was characterized by a specific set of features including marital situation, presence of children at home, same bedtime, shared bedroom, mattress width, number of years together, employment, travel time for work, age, and gender (Table 2). The resulting couple clusters identified were: cluster 1, older retired couples (*n* = 76, 26%); cluster 2, young working couples (*n* = 128, 44%); and cluster 3, mature active couples (*n* = 86, 30%; Figure 1).

### 3.3. Baseline Couple Cluster Data

#### 3.3.1. Baseline Epworth Sleepiness Score

The ESS was significantly different according to the couple clusters at baseline. Young working and mature active couples presented a significantly higher ESS score at baseline compared to older retired couples (median ESS for young couples 12 (IQR: 8–16); mature couples 11 (IQR: 7–15) and older retired couples 7 (IQR: 5–12); *p* < 0.01).

#### 3.3.2. Baseline Quebec Sleep Questionnaire Score

The QSQ score was higher for the young working couples cluster compared to the older retired couples cluster only (71 (IQR: 55–83) vs. 60 (IQR: 45–70), respectively, *p* < 0.01).

#### 3.3.3. Baseline Quality Marriage Index

At baseline, the older retired couples cluster had a lower QMI compared to the two other couple clusters (median QMI: 18 (IQR: 16–21) vs. 20 (IQR: 18–22) vs. 20 (IQR: 17–22), in the older retired, young working and mature active couple clusters, respectively, *p* < 0.01).

### 3.4. Impact of Couple Clusters on CPAP Adherence and Questionnaires

#### 3.4.1. CPAP adherence

**Figure 2:**
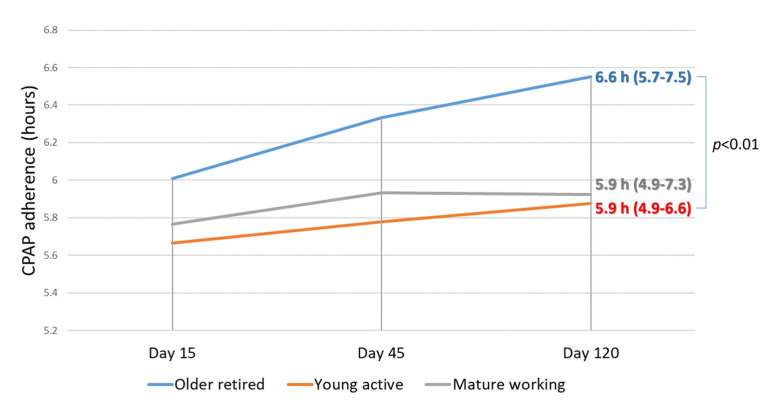
CPAP adherence data in the three couple clusters at day 15, day 45, and day 120 after CPAP initiation.

The primary analysis of the impact of couple clusters on CPAP compliance at day 120 showed a significant difference; the highest CPAP compliance was observed in the cluster of older retired couples compared to the two other clusters (6.6 h (IQR: 5.7–7.5) vs. 5.9 h (IQR: 4.9–6.6) vs. 5.9 h (IQR: 4.9–7.3) for the retired, young and mature couple clusters, respectively, *p* < 0.05; Figure 2). The multivariable mixed linear regression analysis showed that the use of an orofacial mask and couple clusters were associated with CPAP adherence. Compared to the cluster of older retired couples: the cluster of young active couples presented significantly lower CPAP adherence (−30 min (IQR: −54 to −4) *p* = 0.02). Furthermore, the use of an orofacial mask was associated with a significant decrease in 120-day CPAP adherence: 40 min (IQR: −68 to −12; *p* = 0.006). A subgroup analysis in the couples sharing a bedroom (i.e., excluding patients who sleep in separate bedrooms) yielded similar results for CPAP adherence to those obtained when all couples were considered. We also conducted a subgroup analysis in women only. The results were also similar to those obtained when the entire sample was analyzed.

#### 3.4.2. Epworth Sleepiness Score

There was a significant difference in the decrease of the ESS for the cluster of young working and mature active couples compared to the older retired couples cluster. Compared to the older retired couples, the young working couples cluster presented a 2.84-point decrease of the ESS score (IQR: −4.30 to −1.38, *p* < 0.01) and the mature active couples cluster presented a 1.64-point decrease of the ESS score (IQR: −3.2 to −0.05, *p* = 0.04).

#### 3.4.3. Quebec Sleep Questionnaire Score

The overall decrease in the total QSQ score was significantly greater for OSA patients in the cluster of young working couples compared to the clusters of older retired and mature active couples (*p* < 0.01). This was mainly due to a significant decrease in the hyper-somnolence score.

#### 3.4.4. Dyadic Adjustment Scale

There was a significant change in the DAS score between baseline and day-120 between the clusters of older retired couples and young active couples. The median increase in DAS in the cluster of young active was 3 points (IQR: −1.5–7) while the cluster of older retired couples presented a median decrease of −2 points (IQR: −6–6).

#### 3.4.5. Quality of Marriage Index

There was no significant change over time in the QMI in any couple cluster. At day 120 only the difference between the clusters of young working and older retired couples remained significant (i.e., retired couples cluster presented a lower QMI compared to the young couples cluster).

#### 3.4.6. Spouse’s Sleep Quality

The spouse’s sleep quality was lower in the older retired couples cluster compared to the young active couples cluster. Spouses in the retired couples cluster reported a higher level of noise nuisance (moderate level: 3 (IQR: 2–3)) compared to young or mature couples (low level: 2 (IQR: 2–2)).

#### 3.4.7. Spousal Involvement

There were no significant differences between the three couple clusters in term of spousal involvement in the CPAP therapy. There is a slight trend to a higher involvement for spouse’s in the young couples cluster compared to the clusters of mature and retired couples, but this difference was not significant.

## 4. Discussion

In this multicenter prospective study, we used a latent class analysis to identify homogenous groups of spousal relationships. This analysis resulted in the identification of three couple clusters: older retired couples, young working couples, and mature active couples.

Our analysis evaluating the impact of the couple cluster on CPAP adherence showed that the older retired couples cluster presented significantly higher CPAP adherence compared to the two other couple clusters. Interestingly, the older retired couple cluster presented a lower ESS score and an alteration in quality of life related to OSA at baseline compared to the two other couple clusters. Nevertheless, our data are consistent with previous findings showing that increased age is associated with higher adherence to CPAP [24,25] even though other authors have found inconsistent findings [26].

The clusters of young working and mature active couples significantly decreased their ESS score after 120 days of CPAP. Interestingly, the young working couples cluster presented 2.84-point decrease in their ESS score, which is greater than the minimum important difference [27], indicating a clinically significant improvement in this parameter. However, this significantly higher improvement in ESS and QSQ did not translate in a higher CPAP adherence. This demonstrates that CPAP adherence is also impacted by different social activities and constraints during the lifespan including children care, work, sleep quality, and duration.

Accordingly, we observed significant change in the DAS score between baseline and day-120 between the clusters of older retired couples and young active couples: the young active couples increased their DAS by 3 points while the older retired couples presented a 2-point decrease. Furthermore, the sum of DAS scores was lowest in the older retired couples cluster. This was mostly due to differences in the cohesion (i.e., exchanging ideas, engaging in calm discussions…) and consensus scores (i.e., handling finances, social life, life philosophy, major decisions, household tasks…). Thus, despite presenting the highest CPAP adherence, the older retired couples cluster presented the lowest dyadic adjustment. This is in contrast with a previous study in 23 married men with OSA, which found an association between CPAP adherence at three months and perceptions of collaborative spousal involvement [28]. However, spousal involvement was not measured using the DAS, thus rendering our results not entirely comparable.

Another study in which patients reported seeking OSA treatment due to their spouses (rather than being self-referred), demonstrated lower CPAP adherence over the first 120 days of treatment [29]. Furthermore, adherence consistently declined over the follow-up period in spouse-referred patients, whereas CPAP use in self-referred patients remained consistent. The finding that spouse influence was common and was also related to poorer adherence suggests that spouse influence may initially promote treatment initiation, but may also have negative consequences for adherence over time, particularly if at odds with patient preferences or if viewed as coercive.

A recent study examining the role of spousal involvement in CPAP adherence found that spousal involvement is important in determining CPAP compliance in men in the six months after CPAP initiation but does not predict long-term adherence [25]. The authors of this study suggest that involvement of the spouse should be considered an integral part of CPAP initiation procedures. Furthermore, a recent review has highlighted the importance of having a partner involved as a member of the team, as long as a positive relationship exists. This can contribute to producing positive adherence outcomes [30]. While our study focused on the spouse, it is important to note that the influence of a person who can relate to the patient can extend beyond the spouse. For example, a study in which successful CPAP users served as buddies for recently diagnosed OSA patients showed that their support and interaction resulted greater CPAP use after three months of CPAP treatment compared to standard care [31].

We did not observe a significant change in the QMI over time in any of the couple clusters. This is likely due to the short time frame of the study (i.e., 120 days) and the fact that CPAP can only have a limited impact on the quality of marriage. Conversely, McFadyen et al. reported that patients using CPAP demonstrated improved marital satisfaction compared with patients who were treated with weight loss and exercise recommendations and were on a waiting list for CPAP treatment [32]. These results are important for clinicians, as they should report to the patients and spouse only realistic expectations regarding improvement of relationship after CPAP treatment.

This study does have limitations. By requiring consent from both the patient and the partner, this study may have selected couples in more positive relationships. Additionally, our study included a majority of men with OSA. Nevertheless, a subgroup analysis in the women OSA patients yielded similar results to those obtained in the entire sample of patients. Future studies including a larger sample of women are needed. Future investigations should investigate how factors such as disease severity and co-sleeping may influence couples’ experience with CPAP treatment.

## 5. Conclusions

In conclusion, in a large cohort of OSA patients in whom clusters of couples were determined, three couple profiles were identified. There was a significant difference based on CPAP adherence at day-120 after CPAP initiation. The results from the present study highlight the importance of evaluating dyadic coping and marital quality in recently diagnosed OSA patients and including the spouse in a holistic CPAP therapy approach.

## Figures and Tables

**Table 1:** Baseline patient characteristics based on the couples cluster.

Variables	Class 1: Older Retired Couples *n* = 76 (26%)	Class 2: Young Working Couples *n* = 128 (44%)	Class 3: Mature Active Couples *n* = 86 (30%)	Overall *p*-Value
Age (year)	67 (63–71)	45 (40–49)	57 (54–59)	<0.01
Gender (male)	60 (80)	102 (79.7)	62 (72.1)	0.36
BMI (kg/m^2^)	32.1 (27.6–35.7)	32.1 (28.9–35.8)	32.1 (27.7–37.4)	0.81
AHI (events/h)	42 (34.7–52)	43.2 (33–60.1)	44.2 (32–57)	0.89
Epworth score	7 (5–12)	12 (8–16)	11 (7–15)	<0.01
Orofacial CPAP mask (%)	21 (28)	24 (19.5)	18 (21.4)	0.37
APAP (%)	70 (94.6)	120 (95.2)	77 (89.5)	0.23

Results are expressed as median (IQR) or as a percentage (%). Abbreviations: BMI: body mass index; AHI: apnea-hyponea index; CPAP: continuous positive airway pressure; APAP: auto-adjustable positive airway pressure; IQR: inter-quartile range.

**Table 2:** Baseline couple cluster characteristics.

Variables	Variable Items	Class 1: Older Retired Couples *n* = 76 (26%)	Class 2: Young Working Couples *n* = 128 (44%)	Class 3: Mature Active Couples *n* = 86 (30%)	Overall *p*-Value
Marital situation	Living together	9 (12.3)	29 (22.7)	3 (3.5)	<0.01
	Married	64 (87.7)	89 (69.5)	82 (95.3)	
	Civil union	0 (0)	10 (7.8)	1 (1.2)	
Children at home	Yes	11 (15.3)	106 (83.5)	37 (43.5)	<0.01
Same bedtime	Yes	39 (54.9)	60 (47.2)	55 (64)	0.06
Separate bedroom	Yes	22 (29.7)	40 (31.7)	18 (21.7)	0.27
Size of mattress	>140 cm	31 (46.3)	79 (63.7)	46 (59.7)	0.06
Number of years together	10 to 30 years	13 (19.4)	72 (58.5)	25 (29.4)	<0.01
	<10 years	2 (3)	51 (41.5)	5 (5.9)	
	>30 years	52 (77.6)	0 (0)	55 (64.7)	
Professional activity	Both working	0 (0)	96 (75)	43 (53.1)	<0.01
	No one working	73 (97.3)	0 (0)	1 (1.2)	
	One is working	2 (2.7)	32 (25)	37 (45.7)	

Results are expressed as median (IQR).

## References

[1] Benjafield A.V., Ayas N.T., Eastwood P.R., Heinzer R., Ip M.S.M., Morrell M.J., Nunez C.M., Patel S.R., Penzel T., Pépin J.L.D. (2019). Estimation of the global prevalence and burden of obstructive sleep apnoea: A literature-based analysis. Lancet Respir. Med..

[2] Levy P.A., Kohler M., McNicholas W.T., Barbé F., McEvoy R.D., Somers V.K., Lavie L., Pépin J.L. (2015). Obstructive sleep apnoea syndrome. Nat. Rev. Dis. Primers.

[3] Yu J., Zhou Z., McEvoy R.D., Anderson C.S., Rodgers A., Perkovic V., Neal B. (2017). Association of positive airway pressure with cardiovascular events and death in adults with sleep apnea: A systematic review and meta-analysis. JAMA.

[4] Cistulli P.A., Armitstead J., Pepin J.L., Woehrle H., Nunez C.M., Benjafield A., Malhotra A. (2019). Short-term CPAP adherence in obstructive sleep apnea: A big data analysis using real world data. Sleep Med..

[5] Baratta F., Pastori D., Bucci T., Fabiani M., Fabiani V., Brunori M., Loffredo L., Lillo R., Pannitteri G., Angelico F. (2018). Long-term prediction of adherence to continuous positive air pressure therapy for the treatment of moderate/severe obstructive sleep apnea syndrome. Sleep Med..

[6] Chai-Coetzer C.L., Luo Y.M., Antic N.A., Zhang X.L., Chen B.Y., He Q.Y., Heeley E., Huang S.G., Anderson C.S., Zhong N.S. (2013). Predictors of long-term adherence to continuous positive airway pressure therapy in patients with obstructive sleep apnea and cardiovascular disease in the save study. Sleep.

[7] Weaver T.E., Grunstein R.R. (2008). Adherence to continuous positive airway pressure therapy. Proc. Am. Thorac. Soc..

[8] Diaz-Abad M., Chatila W., Lammi M.R., Swift I., D’Alonzo G.E., Krachman S.L. (2014). Determinants of CPAP adherence in hispanics with obstructive sleep apnea. Sleep Disord..

[9] Salepci B., Caglayan B., Kiral N., Sarac G., Fidan A., Torun E., Comert S.S., Gungor G.A. (2013). CPAP Adherence of Patients With Obstructive Sleep Apnea. Respir. Care.

[10] Weaver T.E., Maislin G., Dinges D.F., Bloxham T., George C.F.P., Greenberg H., Kader G., Mahowald M., Younger J., Pack A.I. (2007). Relationship between hours of cpap use and achieving normal levels of sleepiness and daily functioning. Sleep.

[11] Berg C.A., Upchurch R. (2007). A developmental-contextual model of couples coping with chronic illness across the adult life span. Psychol. Bull..

[12] Broström A., Nilsen P., Johansson P., Ulander M., Strömberg A., Svanborg E., Fridlund B. (2010). Putative facilitators and barriers for adherence to CPAP treatment in patients with obstructive sleep apnea syndrome: A qualitative content analysis. Sleep Med..

[13] Gentina T., Bailly S., Jounieaux F., Verkindre C., Broussier P.M., Guffroy D., Prigent A., Gres J.J., Kabbani J., Kedziora L. (2019). Marital quality, partner’s engagement and continuous positive airway pressure adherence in obstructive sleep apnea. Sleep Med..

[14] Merenda A., Miano P. (2015). Co-parental couples and new families: A study of the primary triad. Procedia Soc. Behav. Sci..

[15] Berry R.B., Budhiraja R., Gottlieb D.J., Gozal D., Iber C., Kapur V., Marcus C.L., Mehra R., Parthasarathy S., Quan S.F. (2012). Rules for scoring respiratory events in sleep: Update of the 2007 AASM manual for the scoring of sleep and associated events. J. Clin. Sleep Med..

[16] Lacasse Y., Bureau M.P., Series F. (2004). A new standardised and self-administered quality of life questionnaire specific to obstructive sleep apnoea. Thorax.

[17] Norton R. (1983). Measuring marital quality: A critical look at the dependent variable. J. Marriage Fam..

[18] Carey M.P., Spector I.P., Lantinga L.J., Krauss D.J. (1993). Reliability of the dyadic adjustment scale. Psychol. Assess..

[19] Lewis K.E., Seale L., Bartle I.E., Watkins A.J., Ebden P. (2004). Early predictors of CPAP use for the treatment of obstructive sleep apnea. Sleep.

[20] Baron K.G., Smith T.W., Berg C., Czajkowski L.A., Gunn H., Jones C.R. (2010). Spousal involvement in CPAP adherence among patients with obstructive sleep apnea. Sleep Breath..

[21] Wiese H.J., Boethel C., Phillips B., Wilson J.F., Peters J., Viggiano T. (2005). CPAP compliance: Video education may help!. Sleep Med..

[22] Hagenaars J.A., McCutcheon A.L. (2002). Applied Latent Class. Analysis.

[23] Marbac M., Sedki M. (2019). VarSelLCM: An R/C++ package for variable selection in model-based clustering of mixed-data with missing values. Bioinformatics.

[24] Sin D., Mayers I., Man G.C.W., Pawluk L. (2002). Long-term compliance rates to continuous positive airway pressure in obstructive sleep apnea. Chest.

[25] Batool-Anwar S., Baldwin C.M., Fass S., Quan S.F. (2017). Role of spousal involvement in continuous positive airway pressure (CPAP) adherence in patients with obstructive sleep apnea (OSA). Southwest J. Pulm. Crit. Care.

[26] McArdle N., Kingshott R., Engleman H.M., Mackay T.W., Douglas N.J. (2001). Partners of patients with sleep apnoea/hypopnoea syndrome: Effect of CPAP treatment on sleep quality and quality of life. Thorax.

[27] Crook S., Sievi N.A., Bloch K.E., Stradling J.R., Frei A., Puhan M.A., Kohler M. (2019). Minimum important difference of the Epworth Sleepiness Scale in obstructive sleep apnoea: Estimation from three randomised controlled trials. Thorax.

[28] Baron K.G., Gunn H.E., Czajkowski L.A., Smith T.W., Jones C.R. (2012). Spousal involvement in CPAP: Does pressure help?. J. Clin. Sleep Med..

[29] Hoy C.J., Vennelle M., Kingshott R.N., Engleman H.M., Douglas N.J. (1999). Can intensive support improve continuous positive airway pressure use in patients with the sleep apnea/hypopnea syndrome?. Am. J. Respir. Crit. Care Med..

[30] Weaver T.E. (2019). Novel aspects of CPAP treatment and interventions to improve CPAP adherence. J. Clin. Med..

[31] Parthasarathy S., Wendel C., Haynes P.L., Atwood C., Kuna S. (2013). A pilot study of CPAP adherence promotion by peer buddies with sleep apnea. J. Clin. Sleep Med..

[32] McFadyen T.A., Espie C.A., McArdle N., Douglas N.J., Engleman H.M. (2001). Controlled, prospective trial of psychosocial function before and after continuous positive airway pressure therapy. Eur. Respir. J..

